# Smartphone-based device for point-of-care diagnostics of pulmonary inflammation using convolutional neural networks (CNNs)

**DOI:** 10.1038/s41598-024-54939-4

**Published:** 2024-03-22

**Authors:** Mohammadreza Ghaderinia, Hamed Abadijoo, Ashkan Mahdavian, Ebrahim Kousha, Reyhaneh Shakibi, S. Mohammad-Reza Taheri, Hossein Simaee, Ali Khatibi, Ali Akbar Moosavi-Movahedi, Mohammad Ali Khayamian

**Affiliations:** 1 Institute of Biochemistry and Biophysics, University of Tehran, Tehran, 1417614335 Iran; 2 Integrated Biophysics and Bioengineering Lab (iBL), Institute of Biochemistry and Biophysics, University of Tehran, Tehran, 1417614335 Iran; 3 Nano Electronic Center of Excellence, Nano Bio Electronics Devices Lab, School of Electrical and Computer Engineering, University of Tehran, P.O. Box 14395/515, Tehran, Iran; 4 Department of Biophysics, Faculty of Biological Sciences, Tarbiat Modares University, Tehran, Iran; 5 Groningen university, University medical center Groningen, Antonius Deusinglaan 1, 9713AW Groningen, The Netherlands; 6 Condensed Matter National Laboratory, Institute for Research in Fundamental Sciences (IPM), Tehran, Iran; 7 Department of Biotechnology, Faculty of Biological Sciences, Alzahra University, Tehran, Iran; 8 Cardiac Primary Prevention Research Center, Cardiovascular Diseases Research Institute, Tehran University of Medical Sciences, Tehran, Iran

**Keywords:** Biomedical engineering, Electrical and electronic engineering, Translational research

## Abstract

In pulmonary inflammation diseases, like COVID-19, lung involvement and inflammation determine the treatment regime. Respiratory inflammation is typically arisen due to the cytokine storm and the leakage of the vessels for immune cells recruitment. Currently, such a situation is detected by the clinical judgment of a specialist or precisely by a chest CT scan. However, the lack of accessibility to the CT machines in many poor medical centers as well as its expensive service, demands more accessible methods for fast and cheap detection of lung inflammation. Here, we have introduced a novel method for tracing the inflammation and lung involvement in patients with pulmonary inflammation, such as COVID-19, by a simple electrolyte detection in their sputum samples. The presence of the electrolyte in the sputum sample results in the fern-like structures after air-drying. These fern patterns are different in the CT positive and negative cases that are detected by an AI application on a smartphone and using a low-cost and portable mini-microscope. Evaluating 160 patient-derived sputum sample images, this method demonstrated an interesting accuracy of 95%, as confirmed by CT-scan results. This finding suggests that the method has the potential to serve as a promising and reliable approach for recognizing lung inflammatory diseases, such as COVID-19.

## Introduction

The emergence of the SARS-CoV-2 virus and the subsequent outbreak of one of the most lethal pandemics of the current century^1^, led to the development of advanced technologies for the early diagnosis of COVID-19^2–6^. Various state-of-the-art solutions, including but not limited to ID NOW^7^, RDSS^8^, and others^9–11^, have been introduced to address the urgent need for accurate and rapid point-of-care (POC) diagnostics due to the multiple mutations in the virus^12^. Such developments aim to alleviate the strain on medical centers and enable individuals to conduct COVID-19 tests conveniently at home using rapid, reliable, and smartphone-based approaches^13,14^. However, there remains a need for rapid point-of-care diagnostic approaches that can be conveniently used outside laboratory settings to keep pace with the ongoing pandemic^15^.

While smartphone-based diagnostic devices have been a subject of research for years^16,17^, the convergence of artificial intelligence (AI) and portable systems, particularly smartphones, presents a compelling avenue for rapid POC diagnostics.

AI, as an accepted and popular method, has been recently employed for big data analysis not only for tackling COVID-19 pandemic^18^ and its management^19,20^ but also in different medical fields such as cancer pathology^21,22^, ovulation prediction^23^, X-ray image analysis^24–26^, and etc^27,28^. Compared to a human, AI-based devices are able to analyze the data with superior precision and accuracy. In particular, the use of convolutional neural networks (CNNs) in these devices has shown great promise for detecting various diseases. CNNs are a type of deep learning algorithm capable of recognizing patterns in images and other complex data types^29^. Researchers have recently explored the potential of smartphone-based devices and CNNs for point-of-care diagnostics of pulmonary inflammation^30,31^.

Using AI, lung-related diseases have been classified and detected in several studies^28^. With the use of an electronic stethoscope, Aykanat et al. devised a non-invasive method to classify respiratory sounds^32^. In their study, they found that CNNs and support vector machines (SVMs) accurately classified respiratory sounds. Through the use of high-resolution CT images and deep learning, Chen et al. developed an engine for detecting COVID-19 disease^33^. Deep learning model performance was comparable with that of expert radiologists, thereby improving radiologists' clinical efficiency. A transfer learning approach with deep CNNs was used by Eman et al. to determine which cases were tuberculosis and which were normal from chest radiographs^34^. According to David et al., lateral flow rapid diagnostic tests (RDTs) for SARS-CoV-2 can be read and interpreted with the help of a smartphone application. Especially for prospective validation of real-life scenarios, as well as for antibody, antigen and peptide detection tests, their AI algorithm has demonstrated excellent performance^35^. A smartphone-based breathing sound indicator may be a promising marker for COVID-19 cases, according to Mohanad et al. Based on their study, deep learning can be used as a pre-screening tool before RT-PCR, which is considered the gold standard in such cases^36^. However, smartphone-assisted AI diagnostics have not been explored in the context of detecting COVID-19 lung inflammation from sputum samples.

Here, we aim to bridge this technology gap by introducing a smartphone-based AI framework to trace pulmonary inflammation in COVID-19 cases by ferning pattern analysis of air-dried sputum samples using a low-cost, smartphone-compatible microscopy device. This paper presents the demonstration of an accessible deep learning-powered mobile platform for diagnosing respiratory inflammation without requiring CT infrastructure or extensive sample analysis. Our approach could allow rapid screening of COVID-19 positive patients to determine disease severity and need for intervention.

During the immunological phase of COVID-19, the lungs become inflamed as immune cells battle the virus^37^. This recruits additional immune cells, often disrupting blood vessels and increasing vascular permeability that allows fluid to fill the alveoli^38,39^. Currently, a chest CT scan diagnoses such inflammation. The inflamed state allows blood components like electrolytes to emerge in patient sputum. Thus, increased blood markers in respiratory secretions may indicate lung inflammation. Body fluids, especially blood, have long provided diagnostic indicators^40–42^, aside from its use of treatment purposes^40,43,44^. Various blood-related molecules found in sputum now serve as disease biomarkers, such as glucose in pneumonia^45,46^, salivary components for autoimmune diseases^47^, etc. Sputum electrolyte levels have also been exploited^48,49^, as salt crystals such as sodium chloride (NaCl) can create fern-like sputum patterns that associate with ovulation timing^23^ and conditions like cancer^49^ and Sjogren syndrome^50^. Despite using saliva/sputum to detect COVID-19, no smartphone-assisted deep learning approach has leveraged rising sputum electrolytes and fern-like patterns to trace COVID-19 inflammation. This capability could improve screening for severe lung involvement.

A patient's risk of severe COVID-19 rises with greater immune response, increasing chances of death from lung inflammation. Thus, rapidly diagnosing inflammation could improve outcomes by enabling earlier intervention. In this study, we utilized sputum samples from patients with positive lung CT scans as an indicator of pulmonary inflammation. We examined the fern patterns in their sputum using an affordable, simple, and portable smartphone-based device. In 10 μL sputum samples from CT-positive patients, these patterns were considered as biomarkers of inflammation. First, elevated sputum electrolytes (sodium, potassium) were confirmed in COVID-19 patients using standard analysis, agreeing with our vascular permeability hypothesis. Next, AI-based evaluation of smartphone-captured images showed this approach accurately diagnosed 160 patient-derived sputum sample images with 95% accuracy compared to CT-scan inflammation signs. This compact, low-cost methodology could aid the screening of any lung inflammatory diseases like COVID-19 and facilitate treatment decisions through point-of-care assessment of lung involvement.

## Materials and methods

### Experimental setup and sputum collection

COVID-19 patients suspected of pulmonary involvement, referred by their doctors for lung CT imaging, were selected and divided into two cohorts based on positive and negative CT results. A positive CT scan indicated the presence of common patterns such as ground glass opacification (GGO), consolidation, hazy patches, etc^51^. Sputum samples were collected from both groups, comprising a total of 70 participants, all of whom were duly informed and provided consent for the research. The sputum collection took place early in the morning, with volunteers fasting, and individuals with a history of tobacco and alcohol consumption were excluded from the cohort to prevent fern-like patterns unrelated to inflammation. Following collection, the sputum was left undisturbed for 30 min to allow for the precipitation of cells and other residues.

A portion of the collected sputum was used for electrolyte measurement, and a small volume (10 µl) was deposited on the surface of a sample slide, left at room temperature for air drying. Subsequently, the dried sample was inserted into the mini-microscope to capture fern patterns, which were then analyzed using an AI system.

### Mini-microscope system

To capture fern patterns with the desired resolution using a smartphone camera, we employed a smartphone-based microscopy tool measuring 60 × 60 × 60 mm. The microscope structure was meticulously designed using SOLIDWORKS (Dassault Systèmes) and 3D printed from polylactic acid (PLA) using Ultimaker 2+. The imaging system comprises a plano-convex lens with a 5 mm diameter and a 6 mm focal length serving as an objective lens. Additionally, a commercial acrylic condenser lens compatible with the chosen light-emitting diode was incorporated. Illumination was provided by a 3 V and 5 W white LED, powered by a CR2032 battery (Camelion) series with a 1.2 kΩ resistor to achieve optimal light intensity. The entire electronic circuit is integrated into a Printed Circuit Board, with all lenses, including the smartphone lens and the LED, sharing a common optical axis.

The optical system's spacings were meticulously designed to achieve a 40× magnification within a 5 × 5 mm field of view. This design allows the visualization and processing of the entire fern-containing droplet within the field of view. To activate the illumination system and position the fern sample within the field of view zone, the glass slide containing the fern pattern is inserted into the imaging system. The fern sample is optimally focused within the working distance of the optical system, mitigating the need for manual focusing and minimizing perturbations caused by the user. It's worth noting that the auto-focus feature of the smartphone camera compensates for variations in the optical properties of different smartphones.

### AI algorithm development

To detect ferning patterns in saliva on a smartphone, we used EfficentNet-B0 architecture. This model showed a top-1 accuracy of 77.3% and a top-5 accuracy of 93.5%, performing across 1000 classes of the ImageNet database^52^. To achieve this efficiency, EfficientNet B0 employs a compound scaling method to simultaneously adjust its depth, width, and resolution. By using this method, images can be classified more accurately with fewer parameters, resulting in more complex representations. EfficientNet-B0 is a mobile-sized architecture having 5.3 million trainable parameters. Such a complex neural network needs a significant number of images to optimize its parameters while training. Due to our insufficient dataset of 650 images, we utilized "transfer learning" to develop the algorithm. In this regard, EfficientNet-B0 was first pre-trained with 14 million images of ImageNet. ImageNet is an open-source data set containing 14 million classified images from various categories^53^. Then, we retrained and validated the pre-trained model using our dataset of 650 salivary images derived from 70 participants, categorized into ferning and non-ferning groups. The labeled image dataset was split into 80% (520) for training and 20% (130) for validation. Using an 80:20 convention balances providing adequate new sputum images to fine-tune the model for the desired classification task, while retaining sufficient previously “unseen” images to evaluate model performance on distinguishing ferning versus non-ferning patterns and check for overfitting. Other partition ratios (90:10 and 70:30) were tested during development to validate that this 80:20 allocation provided optimal performance. All the input images were resized to 224 × 224 pixels, and the retraining process was done for 80 training steps (epochs) with a learning rate of 0.001. Cross-entropy as a loss function and accuracy was measured to evaluate the learning process of the model. Training and validation accuracy are the percentages of correctly detected images by the model in the training and validation datasets. The variance between training and validation accuracies was calculated to know if the model was overfitting. At the 80th training step (epoch), the validation cross-entropy/loss and accuracy were 0.102and 98.23%, respectively. The weights corresponding to the best performance were saved and used for the model.

The image processing component outside the Convolutional Neural Network (CNN) encompasses a singular step, entailing the resizing of images to dimensions of 224 * 224 pixels. The neural network comprises two principal segments: the first being feature extraction, employing a pretrained EfficientNet, and the second involving the classification aspect, consisting of four dense layers. Within the initial three layers, Rectified Linear Unit (ReLU) activation is applied to mitigate the issue of gradient vanishing, while the output layer employs the softmax activation function to denote the probability of an image belonging to each respective class. For the training procedure, the cross-entropy loss function is selected, assessing the performance of a classification model outputting probability values within the range of 0 to 1, in alignment with the softmax function output.

The optimization algorithm opted for is RMSProp, utilized to minimize the cross-entropy function. The formulation of this optimization process is expounded upon in the subsequent discourse:

1. Rectified Linear Unit (ReLU) Activation Function Formulation:
  $$f\left(x\right)={\text{max}}(0,x)$$
2. Softmax Activation Function Formulation:
  $$softmax{\left(z\right)}_{i}=\frac{{e}^{{z}_{i}}}{\sum_{j=1}^{k}{e}^{{z}_{j}}}$$
  where ${z}_{i}$ is the input to the ith unit, and K is the total number of units in the layer.
3. Cross-Entropy Loss Function Formulation:
  $$CE\left(y,\widehat{y}\right)=-\sum_{i=1}^{N}{y}_{i}.{\text{log}}(\widehat{{y}_{i}})$$
  where N is the number of classes, ${y}_{i}$ is the true probability of class i, and $\widehat{{y}_{i}}$ is the predicted probability of class i.
4. Root Mean Square Propagation (RMSProp) Optimization Algorithm Formulation:
  $${\uptheta }_{t+1} ={\uptheta }_{t} - \frac{\upeta }{\sqrt{{\text{E}}{\left[{{\text{g}}}^{2}\right]}_{t}+\epsilon }}.{{\text{g}}}_{{\text{t}}}$$
  where $\theta$ is the parameter at time t, $\eta$ is the learning rate, ${{\text{g}}}_{{\text{t}}}$ is the gradient at time t, ${\text{E}}{\left[{{\text{g}}}^{2}\right]}_{t}$ is the exponentially weighted moving average of squared gradients up to time t, and $\epsilon$ is a small constant added for numerical stability.

Here is the neat sketch of the whole algorithm:

**Algorithm**: Proposed method for detecting COVID-19 lung inflammation using sputum ferning patterns

**Input**: Sputum images from COVID-19 patients

**Output:** Classification as lung inflammation positive or negative

1. **Collect sputum samples from patients sent for CT scans**
2. **Preprocess Images**
  Resize to 224 × 224 resolution
  Convert to grayscale
  Normalize pixel intensities to [0,1]
  Augment data via horizontal flips, rotations
3. **Construct CNN Architecture**
  Use pre-trained EfficientNet-B0 as base model
  Append classification layers
4. **Prepare Training Data**
  Label images as ferning/non-ferning
  Split images 80/20 into training/validation
5. **Train CNN**
  Train for 80 epochs
  Use cross entropy loss, RMSprop optimization
  Learning rate = 0.001
6. **Assess Performance**
  Evaluate accuracy on validation set
  Check for over/under-fitting
7. **Classify Sputum Images**
  Capture microscope images
  Predict ferning presence
  Relate to lung inflammation

### Algorithm evaluation

To evaluate the algorithm, receiving operative characteristic (ROC) curve was plotted. The output of the trained convolutional neural network is a continuous number between 0 and 1, representing the probability of the image belonging to a specific group. The ROC curve is plotted based on different threshold values to find the best operating point based on the purpose. The area under the ROC curve (AUC) indicates the proposed method’s ability to distinguish between different classes. Additionally, to assess the ability of the retrained model to predict accurately, 160 patient-derived sputum sample images, consisting of both ferning and non-ferning cohorts, were utilized.

### Web application development

A web application was developed to analyze the presence of ferning patterns in salivary images. Initially, a drop of the sputum sample is placed at the center of the sample slide. After air-drying, the slide is inserted into the mini-microscope system. Then, the lens provides enough magnification (40X) to observe ferning patterns through any smartphone camera. Subsequently, images are captured and sent to the developed web application, where AI determines the presence of ferning patterns. Also, the designed web application, featuring a convenient and straightforward graphical user interface, enables users to access previous test records to track their disease progression.

### Statistical analysis

A 2 × 2 confusion matrix was plotted to determine specificity sensitivity, positive predictive value (PPV), and negative predictive value (NPV) in comparison to the CT-scan results as the golden standard for validating pulmonary inflammation.

The obtained data in this study was gathered from at least three independent experiments and analyzed using the statistic software GraphPad Prism 8 and expressed as mean ± standard deviation (SD). Statically significant results were reported when the P values were less than 0.05 for a specific experiment.

### Ethics and consent to participate

All methods employed in our study were conducted in strict accordance with relevant guidelines and regulations imposed by the University of Tehran. Informed consents were obtained for all participants who were made aware of the planned publication through consent forms, and all the experiments were conducted following pertinent guidelines approved by the University of Tehran.

## Results and discussion

### The background biology and microscopic translation of the sputum electrolyte

**Figure 1:**
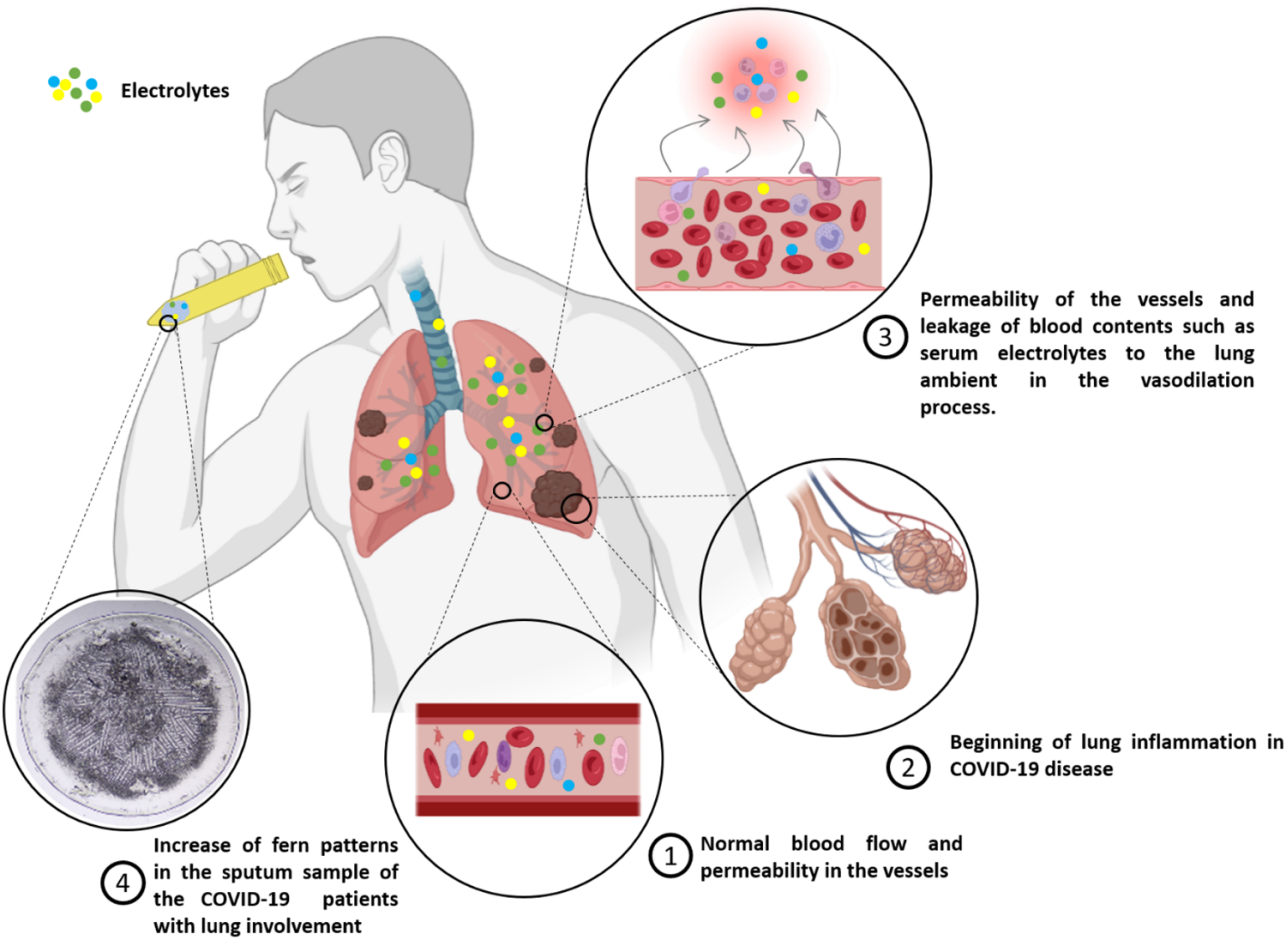
During the inflammation phase of COVID-19, numerous immune cells are mobilized into the lung environment through the vasodilation process. The vessels surrounding the alveoli undergo increased permeability, allowing the entry of blood contents into the lung environment. This phenomenon has the potential to alter the concentration of sputum components, including electrolyte salts.

**Figure 2:**
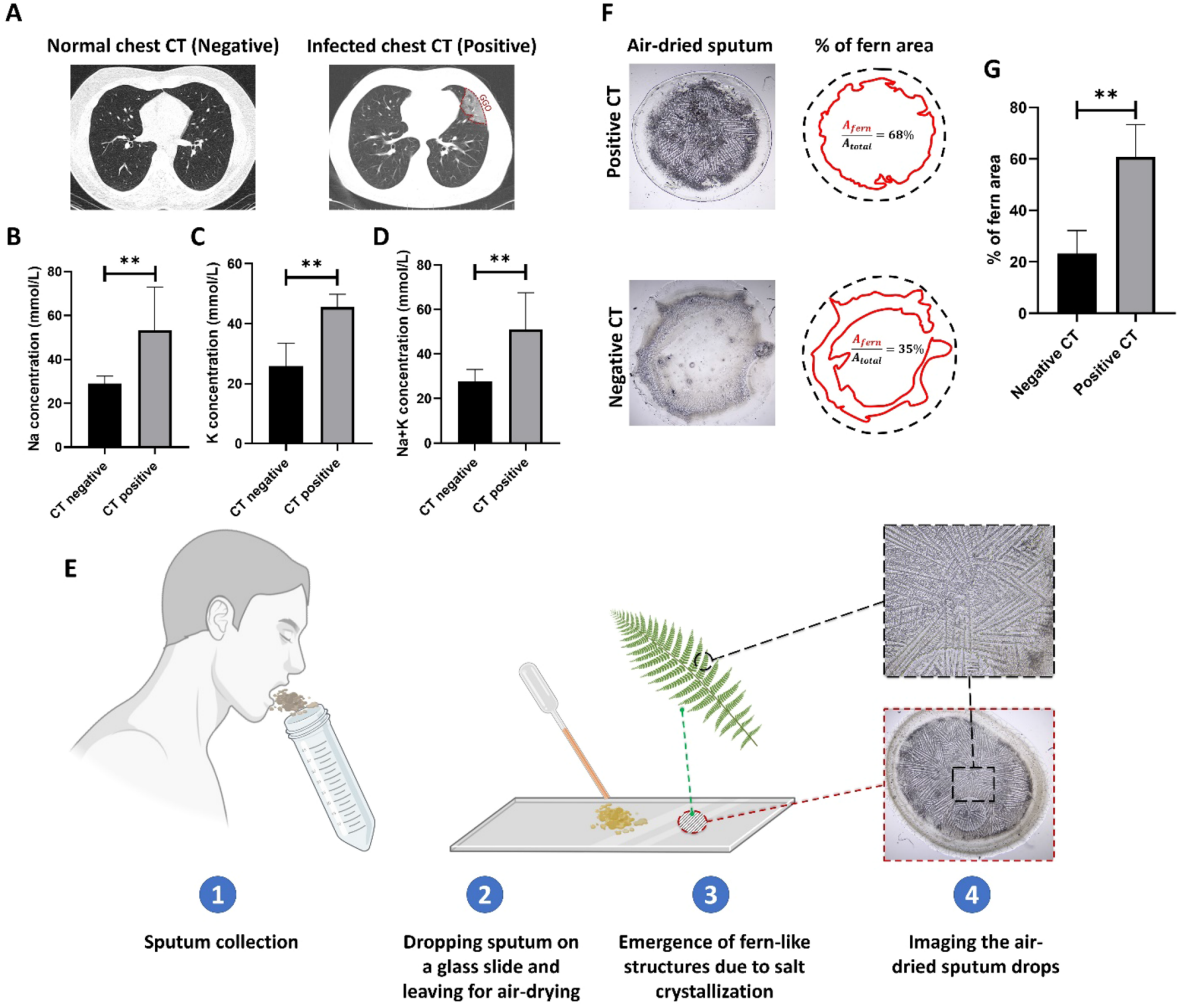
(**A**) CT scan from the two patients with COVID-19 disease and with and without lung inflammation. Signs such as glass ground opacification (GGO) in the CT images imply lung involvement. (**B**–**D**) Display the results of the Na, K, and NA + K concentrations in the negative and positive CT cases, respectively. (**E**) Workflow of the designed method for analyzing the fern structures in the dried sputum samples of the patients with different CT scan results. (**F**) Different ferning patterns in the air-dried samples of the CT positive and negative cases. (**G**) The percentage of the fern area in the sputum images of the CT positive and negative cases.

During the COVID-19 disease, the immune system signals many white blood cells (WBCs) to travel to the lung environment and combat the viruses. To facilitate this process, the tiny micro-vessels around the respiratory alveolus dilate and become permeable, allowing the immune cells to traverse. The increased permeability of the vessels leads to the filling of air sacs with blood fluid, resulting in acute respiratory distress syndrome (ARDS) and subsequent lung failure in certain areas. Our hypothesis posits that blood serum infiltration into the lung environment will alter the composition and concentration of sputum components, particularly electrolyte salts such as Na and K (Fig. 1). Currently, chest CT scans are conducted to monitor inflammation and respiratory involvement in patients. The images with signs of glass-ground opacification (GGO), patchy consolidation, etc., are fallen into this category (Fig. 2A).

Following the CT results, patients were categorized into CT positive and CT negative groups (Fig. 2A). Subsequently, fasting sputum samples were collected from the patients, and an electrolyte analyzer measured their sodium and potassium ions. Sodium and potassium are primary electrolytes in blood serum. Conditions such as hypernatremia and hyperkalemia can contribute to variations in these electrolytes in the blood, potentially leading to various dangerous diseases.

The concentration of the Na and K ions can be observed in Fig. 2B–D. Notably, a significant increase in the concentration of both sodium and potassium is observable in the sputum samples from CT-positive patients. While the average concentration of Na and K in the CT-negative samples is approximately 29 mmol/L and 26 mmol/L, respectively, these values rise to about 53 mmol/L and 46 mmol/L in CT-positive cases, reflecting an increase of approximately 82% and 77%, respectively. These findings support the hypothesis that the electrolytes released into the respiratory system due to inflammation and subsequent vasodilation can be detected in the sputum samples.

As the next step, the increased salt concentration in the sputum was translated into a graphical picture (Fig. 2E). In this regard, a drop of the sputum sample was air-dried on a glass slide and then imaged by a mini-microscope. As shown in Fig. 2F, the branchy and fern-like patterns due to the crystallization of the Na and K salts could be seen in the dried samples. Moreover, in the sputum samples of the CT-positive patients, the fern patterns occupy more area of the whole drop, which confirms the previously obtained results. This number for the patients with negative CT is ~ 23% and ~ 61% for the positive cases (Fig. 2G).

### Design of the mini-microscope

**Figure 3:**
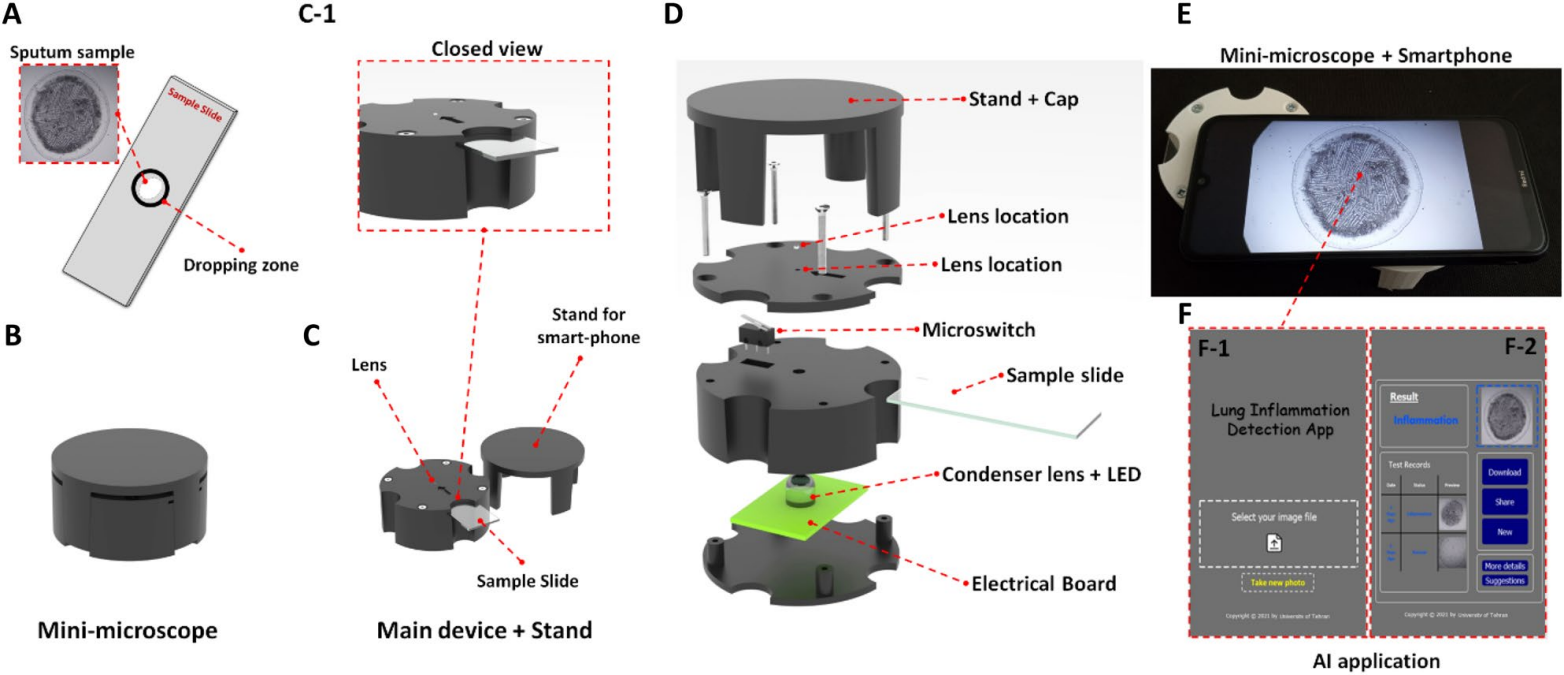
(**A**) Sample slide for dropping the sputum sample (**B**) Mini-microscope system for visualization and imaging of the air-dried sputum sample. (**C**) The main device and closed view (C-1) of the mini-microscope. (**D**) Exploded view of the designed mini-microscope for sputum analysis. (**E**) Placing the smartphone on the mini-microscope and imaging the sputum sample as well as its analysis by the (**F**) AI-based application.

After dropping the sputum sample at the center of the sample slide (Fig. 3A) and allowing it to air-dry, the slide is inserted into the mini-microscope system (Fig. 3B and C) for visualizing the fern patterns. The portable mini-microscope system consists of two main components: a magnifier lens and the lightning board (Fig. 3D). The lens provides a 40× magnification, sufficient to see an air-dried sputum sample with a diameter of 5 mm. The lighting system also consists of an LED attached to an electronic board with a condenser lens for uniform sample illumination. The sample slide is placed between the two lenses in an optimized working distance for having the best focus on the fern structures. A microswitch is also placed for automatic on and off of the system as well as fixing the slide at the location. The cap of the system is designed in a way that could be utilized as the stand for holding the smartphone on top of the mini-microscope (Fig. 3E) and giving the possibility for adjusting the mobile camera with the lens. In the end, the taken image is processed by an AI-based application on the smartphone (Fig. 3F).

### CNN for intelligent detection of the ferning patterns

**Figure 4:**
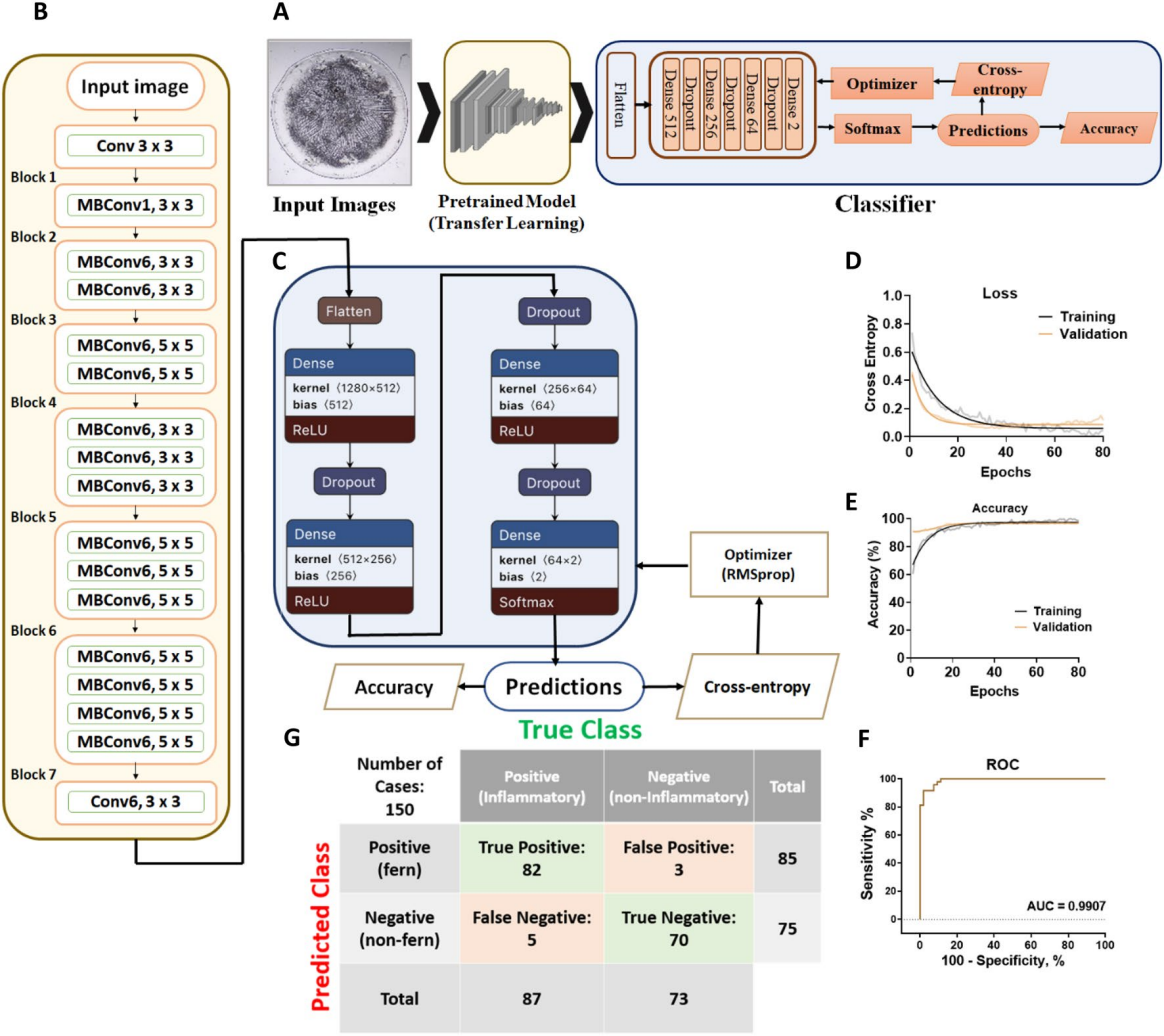
System performance. (**A**) Computational flow of data in the utilized neural network, for transfer learning, the pre-trained EfficientNet was retrained by using our dataset of 650 salivary images derived from 70 participants. (**B**) EfficientNet-B0 structure; this mobile-sized architecture contains 7 main blocks, each containing a varying number of sub-blocks. (**C**) Classifier layers added for retraining and decide about the ferning patterns. (**D**,**E**) training and validation curves for accuracy and cross-entropy of the network; after 80 epochs model achieved a validation accuracy of 98.23% on training set and the validation cross-entropy was 0.18. (**F**) To evaluate the diagnostic ability of the system, receiving operative characteristic (ROC) curve was plotted for different thresholds. The area under the ROC curve (AUC) showed a value of 0.99. (**G**) the confusion matrices for the test sets when smartphone-based device analyzed the patient-derived samples. True classes are determined by CT-scan results.

In this study, we employed artificial intelligence (AI), more precisely Convolutional Neural Networks (CNNs), to detect fern patterns in the sputum of an individual on a smartphone. A CNN is a deep learning model commonly used for computer vision tasks such as image classification, object detection, and segmentation^29^. Through a combination of convolutional layers, pooling layers, and fully connected layers, CNNs are designed to learn hierarchical representations of visual data automatically. Feature extraction is performed by convolutional filters across the input image, while downsampling reduces spatial dimensionality. After the features have been extracted, the fully connected layers map them to the output classes. CNNs have achieved state-of-the-art performance in various computer vision tasks and have become a cornerstone of deep learning research^54^. In this work, EfficientNet-B0 was utilized as a mobile-sized architecture pre-trained with the ImageNet dataset. This pre-trained model was retrained and validated by 650 labeled salivary images gathered from 70 participants (Fig. 4A). Input images are resized to 224 × 224 pixels through the model. The network is uniformly scaled in depth, width, and resolution than conventional structures through the EfficientNet. As shown in (Fig. 4B), this structure contains 7 main blocks, each containing a varying number of sub-blocks, which is followed with the added classifier layers for retraining and decide about the ferning patterns (Fig. 4C).

For transfer learning, the pre-trained EfficientNet was retrained with a learning rate of 0.0001and for 80 training steps (epochs). After 80 steps of training, the model reached an accuracy of 98.23%, while the cross-entropy loss function showed a number of 0.102(Fig. 4D and E). The difference between validation and training accuracies showed that no overfitting had occurred. The weights corresponding to this performance were saved and utilized for the final model. Additionally, the receiving operative characteristic (ROC) curve was plotted for different thresholds to evaluate the system's diagnostic ability. The area under the ROC curve (AUC) showed a value of 0.99, which is highly acceptable (Fig. 4F).

### System performance on patient-derived salivary samples

To evaluate the system's performance in detecting respiratory inflammation through salivary ferning patterns, we gathered 160 saliva sample images out of 70 participants including healthy people. The samples were divided into two cohorts: inflammatory and non-inflammatory. All the samples were tested using our point-of-care smartphone-based AI method, and CT-scan results validated the presence of pulmonary inflammation. Consequently, the 2 × 2 confusion matrix (Fig. 4G) showed an accuracy of 95%. Meanwhile, the specificity and sensitivity were 95.89% and 94.25%, respectively. Further, for this dataset, the proposed method showed a positive predictive value (PPV) of 96.47% and a negative predictive value (NPV) of 93.33% (Table 1).

**Table 1:** Diagnostic parameters of the proposed method (%).

Accuracy	Specificity	Sensitivity	Positive predictive value (PPV)	Negative predictive value (NPV)
95	95.89	94.25	96.47	93.33

## Discussion

The 95% accuracy of this smartphone-assisted AI approach for diagnosing lung inflammatory diseases via sputum analysis shows strong potential as an accessible point-of-care screening tool. Specifically, the ability to rapidly stratify COVID 19 patients based on presence of respiratory involvement could empower quicker treatment decisions and monitoring of disease progression. While CT imaging is the current gold standard, requirements for expensive infrastructure and radiologist availability greatly limit access, whereas this prototype system relies only a miniature microscope and mobile phone.

However, several limitations in the current methodology need to be considered. The dataset comprised a relatively small number of retrospective samples restricted mostly to confirmed COVID-19 cases from a single medical center. Expanding this framework to incorporate various respiratory illnesses and patient demographics could strengthen real-world validity and generalizability. Another limitation of the current smartphone-based AI system is potential interference in identifying lung inflammation for patients with a history of tobacco or alcohol use. The byproducts of smoking and drinking can directly impact sputum composition, including inducing fern-like patterns unrelated to inflammation. Thus, for accurate implementation in the clinical setting, adequate protocols would need to be developed regarding timing of sputum analysis relative to the patient's last smoke or drink. Analyzing samples only after sufficient clearance time of tobacco/alcohol traces would help prevent false positives for pulmonary involvement. Alternatively, collecting data across patient groups with varying smoker/drinker statuses could allow retraining the AI model to account for these factors in its diagnostic algorithm. Implementing such measures to eliminate or adjust for smoking/drinking effects will be important future steps for reducing confounders before widespread adoption.

## Conclusion

In summary, we have introduced a new technique that can serve as an auxiliary method for the current CT scan technique in detecting pulmonary involvement and lung inflammation in patients with conditions like COVID-19. This method has not been applied to other pulmonary diseases such as asthma, COPD, etc. However, given their similar mechanisms to COVID-19, we anticipate that the system will function similarly for them.

The system comprises a portable mini-microscope with 40X optical zoom capability, a glass slide with a designated area for dropping and drying the sputum sample, and an AI-based application on a smartphone for detecting fern patterns on the sputum sample resulting from the crystallization of electrolytes from the blood serum.

To validate our hypothesis regarding the entry of serum electrolytes into the sputum during the inflammation phase of the disease, we initially measured electrolyte concentration (sodium and potassium) in sputum samples from both CT-positive and CT-negative cases. The results ultimately proved our hypothesis that the electrolyte amount is higher in the sputum of CT-positive patients. After that, the fern structures due to the crystallization of the sputum salts were assessed by the mini-microscope with the assistance of a smartphone with an AI-based application. In this regard, the images are sent to a smartphone application to be analyzed using a CNN to detect fern patterns in sputum as a sign of pulmonary inflammation. EfficientNet-B0 was utilized as a mobile-sized CNN architecture, pre-trained with the ImageNet dataset. Using transfer learning, this pre-trained model was retrained and validated by 650 labeled salivary images gathered from 70 participants. Evaluating on 160 patient-derived sputum samples, this method showed a noteworthy accuracy of 95% confirmed by CT-scan results.

Consequently, the study proposes a novel method for detecting inflammation and lung involvement in COVID-19 patients by identifying fern patterns in their sputum samples. An affordable and portable mini-microscope can be used to detect fern patterns in sputum samples using this method, which makes it simple and cost-effective. Using AI to detect the fern patterns, this smartphone application can differentiate between CT positive and negative cases. This method could potentially provide an early indication of lung involvement in COVID-19 patients, allowing for earlier intervention and treatment. We believe this portable chip system could be reliable assistance for doctors, especially in medical centers where CT scan facilities are not provided.

## Data Availability

The datasets used and/or analyzed during the current study are available from the corresponding author upon reasonable request.
