# Author Correction: Smartphone-based device for point-of-care diagnostics of pulmonary inflammation using convolutional neural networks (CNNs)

**DOI:** 10.1038/s41598-024-62914-2

**Published:** 2024-05-31

**Authors:** Mohammadreza Ghaderinia, Hamed Abadijoo, Ashkan Mahdavian, Ebrahim Kousha, Reyhaneh Shakibi, S. Mohammad-Reza Taheri, Hossein Simaee, Ali Khatibi, Ali Akbar Moosavi-Movahedi, Mohammad Ali Khayamian

**Affiliations:** 1 Institute of Biochemistry and Biophysics, University of Tehran, Tehran, 1417614335 Iran; 2 Integrated Biophysics and Bioengineering Lab (iBL), Institute of Biochemistry and Biophysics, University of Tehran, Tehran, 1417614335 Iran; 3 Nano Electronic Center of Excellence, Nano Bio Electronics Devices Lab, School of Electrical and Computer Engineering, University of Tehran, P.O. Box 14395/515, Tehran, Iran; 4 Department of Biophysics, Faculty of Biological Sciences, Tarbiat Modares University, Tehran, Iran; 5 Groningen university, University medical center Groningen, Antonius Deusinglaan 1, 9713AW Groningen, The Netherlands; 6 Condensed Matter National Laboratory, Institute for Research in Fundamental Sciences (IPM), Tehran, Iran; 7 Department of Biotechnology, Faculty of Biological Sciences, Alzahra University, Tehran, Iran; 8 Cardiac Primary Prevention Research Center, Cardiovascular Diseases Research Institute, Tehran University of Medical Sciences, Tehran, Iran

Correction to: *Scientific Reports* 10.1038/s41598-024-54939-4, published online 22 March 2024

The original version of this Article contained an error in the author name S. Mohammad-Reza Taheri which was incorrectly given as Seyed Mohammad Reza Taheri.

In addition, an affiliation was omitted for S. Mohammad-Reza Taheri. The correct affiliations for S. Mohammad-Reza Taheri are listed below.

Groningen University, University Medical Center groningen, Antonius Deusinglaan 1, 9713AW Groningen, the Netherlands.

Condensed Matter National Laboratory, Institute for Research in Fundamental Sciences (IPM), Tehran, Iran.

Moreover, Hossein Simaee was incorrectly affiliated with ‘Institute of Biochemistry and Biophysics, University of Tehran, Tehran 1417614335, Iran’ and ‘Integrated Biophysics and Bioengineering Lab (iBL), Institute of Biochemistry and Biophysics, University of Tehran, Tehran 1417614335, Iran’. The correct affiliation for Hossein Simaee is listed below.

Cardiac Primary Prevention Research Center, Cardiovascular Diseases Research Institute, Tehran University of Medical Sciences, Tehran, Iran.

The original Article has been corrected.

